# Single-nucleus and spatial transcriptomics reveal intestinal cellular heterogeneity, differentiation, and cell communication mechanisms in SAP-induced intestinal injury

**DOI:** 10.3389/fimmu.2026.1719902

**Published:** 2026-01-30

**Authors:** Lechang Zhang, Changqin Xu, Tong Su, Tong Xiao, Xu Yi, Yuemin Feng, Jing Wang, Shulei Zhao

**Affiliations:** Department of Gastroenterology, Shandong Provincial Hospital Affiliated to Shandong First Medical University, Jinan, Shandong, China

**Keywords:** acute pancreatitis, cell communication, cellular heterogeneity, intestinal injury, single-nucleus transcriptomics, spatial transcriptomics, stem cell differentiation

## Abstract

**Background:**

Acute pancreatitis (AP) ranges from mild to severe, and severe disease frequently causes multi-organ damage, with intestinal injury being a major complication. The mechanisms underlying SAP-induced intestinal injury remain unclear, particularly regarding spatial cellular reorganization and functional interactions.

**Methods:**

This study constructed a severe acute pancreatitis (SAP) rat model and employed single-nucleus RNA sequencing (snRNA-seq) and spatial transcriptome sequencing (stRNA-seq) technologies to systematically analyze the dynamic changes in intestinal cellular composition, spatial distribution, and function during SAP-induced intestinal injury.

**Results:**

snRNA-seq identified 18 major ileal cell populations spanning epithelial, immune, stem/TA, and stromal compartments. SAP was associated with compositional remodeling characterized by downward trends in Lgr5^+^/Olfm4^+^ stem cells, TA1, goblet cells, and Paneth cells, with a reciprocal increase in enterocytes, although most proportion changes did not reach statistical significance at the animal level. Spatial transcriptomics independently captured SAP-associated tissue remodeling, including a significant reduction in Paneth cells accompanied by increases in fat cells, macrophages, goblet cells, and TA2 cells. Across epithelial lineages, SAP induced a transcriptional shift toward immune interaction with up-regulation of antigen presentation–related genes (e.g., Cd74) and down-regulation of antimicrobial/barrier effectors (e.g., Defa24, Pla2g2a, Dmbt1), which was corroborated by spatial expression patterns and spatially variable gene programs enriched for host defense responses. Pseudotime analysis suggested a redistribution of epithelial states along the stem/TA-to-enterocyte continuum, with relative depletion of early states and expansion of enterocyte-dominant states in SAP. CellChat analysis revealed globally intensified intercellular communication and nominated FN1 as the pathway with the highest differential information flow, with Lgr5^+^ stem cells predicted as prominent FN1 senders targeting enterocytes and smooth muscle cells. SCENIC identified reduced activity and expression of Hmga2/Myb regulons in stem compartments, and immunofluorescence showed decreasing trends in Hmga2/Myb-positive Olfm4^+^ and Lgr5^+^ stem cells in SAP.

**Conclusions:**

Integrated single-nucleus and spatial transcriptomics reveal that SAP is accompanied by spatially organized ileal remodeling, epithelial immune-interacting rewiring, and altered neighborhood architecture, together with an ECM-centered FN1 signaling axis and attenuated Hmga2/Myb-associated regulatory programs in stem compartments. These findings provide a spatially informed cellular framework and generate testable hypotheses for mechanisms underlying impaired epithelial regeneration during SAP-associated intestinal injury.

## 1 Introduction

Acute pancreatitis (AP) is an unpredictable and potentially life-threatening disease whose global incidence continues to rise annually. Despite advances in supportive treatment and critical care, severe acute pancreatitis (SAP) remains associated with substantial mortality rates (1–3). AP frequently induces multi-organ dysfunction, with the intestine recognized as a primary target organ. Disruption of the intestinal mucosal barrier not only exacerbates local inflammation and disease severity but also precipitates systemic complications. Within this barrier, the intestinal mucosal immune component plays a crucial role, and its aberrant activation drives sustained pro-inflammatory responses, dysregulated intercellular communication, and bacterial translocation (4–6).

The mechanisms underlying intestinal mucosal barrier injury following AP are complex. Multiple cell types, including intestinal epithelial cells, infiltrating immune cells, and intestinal stem cells, contribute to both tissue injury and subsequent repair during AP (7–9). Moreover, these cells exhibit pronounced cellular heterogeneity, assuming distinct functional roles during intestinal injury and pathogenesis (10–14). Epithelial metabolic dysfunction contributes to barrier collapse, as inhibition of intestinal epithelial Pck1 alleviates AP by restoring intestinal homeostasis (9). Microbiota–epithelium interactions are also pivotal: TLR4 deficiency in intestinal epithelial cells exacerbates pancreatic and intestinal injury by inducing gut dysbiosis, particularly through depletion of *Lactobacillus* species, whereas *Lactobacillus reuteri* can maintain intestinal homeostasis and alleviate AP by modulating Paneth cells, which in turn regulate gut microbial composition (15, 16). Furthermore, immune dysregulation contributes to barrier breakdown; aberrant activation of regulatory T cells (Tregs) compromises duodenal barrier integrity, facilitating commensal bacterial translocation and worsening pancreatic necrosis, while S1PR2 contributes to intestinal injury in SAP by driving macrophage pyroptosis (7, 8). Collectively, these findings highlight that AP-induced intestinal barrier injury results from intricate crosstalk among epithelial, microbial, and immune components.

In recent years, single-nucleus RNA sequencing (snRNA-seq) has emerged as a powerful tool for dissecting the cellular landscape of intestinal tissues, enabling high-resolution analysis of disease mechanisms, therapeutic target discovery, and treatment development (17, 18). For example, snRNA-seq of ileal tissues from AP-induced injury and post-treatment rat models has revealed substantial cellular heterogeneity and suggested that fibroblasts participate in intestinal injury and repair through interactions with other cell types (19). Similarly, snRNA-seq of small intestinal tissues at 0–12 hours post-AP induction uncovered dynamic early injury responses and identified mast cell-derived CCL5 as a potential therapeutic target for AP-associated intestinal dysfunction (20). However, while these studies have characterized cellular heterogeneity, they lack spatial context—even though spatial microenvironments, defined by gradients of oxygen, nutrients, extracellular matrix, morphogens, and microbiota, critically influence cellular states and functions. Understanding how the same cell type exhibits functional heterogeneity across distinct spatial niches (e.g., epithelium versus lamina propria) is essential for decoding intestinal pathophysiology (14, 21). Spatial transcriptomics (stRNA-seq) now provides such context, revealing, for example, that goblet and tuft cells at villus tips express high levels of immunoregulatory genes (22), intestinal tissue macrophages display striking spatial diversity (23), and epithelial stem cell proliferation is enriched in regions with reduced p53 activation during intestinal repair (24).

Despite these advances, how intestinal cells are spatially reshaped and how such reorganization translates into functional heterogeneity during SAP-driven injury remain insufficiently defined. Here, we combined a rat SAP model with integrated single-cell and spatial transcriptomics to map the intestinal cellular landscape, architecture, and intercellular circuitry under injured conditions. We specifically asked whether stem-cell pools are quantitatively or qualitatively reshaped, how differentiation trajectories and cell–cell communication networks are modulated, and to what extent non-immune populations participate in immune modulation within the damaged mucosa. Exploring the signalling axes that might govern these responses may refine our mechanistic understanding of SAP-associated barrier failure and highlight previously unrecognized therapeutic targets.

## 2 Methods

### 2.1 Experimental model and animal details

Eight male Sprague–Dawley rats (6 weeks old, SPF grade) were obtained from Wuhan Hualianke Biological Technology Co., Ltd. Animals were housed under SPF conditions (temperature 22–26 °C, relative humidity 50–60%, 12 h/12 h light/dark cycle) and acclimatized for 7 days prior to experimentation. After anesthesia with 3% pentobarbital sodium (40 mg/kg, intraperitoneal injection), a midline laparotomy was performed to expose the pancreaticobiliary duct. Retrograde puncture was conducted using a No. 5 puncture needle (5#) near the duodenal papilla, and 3% sodium taurocholate solution (0.1 mL/100 g body weight) was infused into the pancreatic duct at a rate of 0.1 mL/min with a microinjection pump to induce severe acute pancreatitis (SAP). Following injection, the duct was clamped for 5 min before needle removal and closure of the duodenal wall. Control rats underwent sham laparotomy without infusion. Postoperatively, rats were allowed free access to water but were fasted from food. Animals were divided into control (n=3) and model (n=3) groups, with two additional rats kept as backups.

All animals were sacrificed 24 h after SAP induction, a time point at which SAP-associated intestinal injury is typically evident. At sacrifice, rats were anesthetized with 3% pentobarbital sodium (40 mg/kg, intraperitoneal), and blood was collected from the abdominal aorta. Ileal tissues were rapidly harvested, washed in ice-cold PBS, and flash-frozen in liquid nitrogen for downstream assays. During ileal tissue collection, attached mesenteric fat was trimmed away, segments containing visible Peyer’s patches/lymphoid aggregates were avoided or excised, and the tissue was opened longitudinally and repeatedly rinsed with ice-cold PBS before downstream processing. If animals were not moribund at the endpoint, euthanasia was performed by an overdose of pentobarbital sodium (100 mg/kg, intraperitoneal), and death was confirmed by the absence of heartbeat and respiration.

For validation assays (ELISA, qPCR, Western blot) and snRNA-seq, all six rats (n=3 control, n=3 SAP) were used, whereas for Stereo-seq, four high-quality samples (n=2 control, n=2 SAP) were selected from these animals based on tissue integrity and RNA quality. All experimental procedures involving animals were reviewed and approved by the Shandong Provincial Hospital Committee on Use and Care of Animals (Approval No.: NSFC: No.2025–460) and conducted in accordance with institutional and national guidelines for the care and use of laboratory animals.

### 2.2 Nuclear isolation and quality control

100 mg of tissue homogenate was collected, nuclei were extracted according to the instructions of the Boyou^®^ Cell Nucleus Isolation Kit (SHBIO, 52009-10, China), and the nuclei were counted and evaluated using a fluorescence cell analyzer (Countstar^®^ Rigel S2 (Wuhan)/Mira FL (Northwest China), ALIT Life Science, China) and an inverted microscope for cell nuclear morphology evaluation.

### 2.3 snRNA-seq library preparation and sequencing

Single-nucleus suspensions were prepared using the DNBelab C High-throughput Single-cell RNA Library Preparation Kit V3.0 (MGI Tech). The workflow proceeded as follows: Isolated single nuclei were co-encapsulated with barcoded gel beads and reverse transcriptase mix into oil-in-water droplets using the DNBelab C-TaiM 4 system (MGI Tech). Following cDNA synthesis within the droplets, the emulsions were disrupted. The resultant cDNA was purified, then amplified via PCR using reagents from the DNBelab C High-throughput Single-cell RNA Library Preparation Kit V3.0 (MGI Tech). Amplified cDNA was purified with SPRIselect magnetic beads (Beckman Coulter) according to standard protocols. Subsequent steps included cDNA fragmentation, end repair, and A-tailing, followed by ligation with dual adapters using the DNBelab C Single-cell Library Preparation Sample Labelling Kit S (MGI Tech). The ligated products were purified, subjected to size selection (300–600 bp), amplified, and re-purified. After quantification with a Qubit 4.0 fluorometer, the libraries were stored at -80 °C until sequencing. For high-throughput sequencing, library preparation strictly followed the manufacturer’s instructions, and the final libraries were loaded onto the DNBSEQ-T7 platform for 150 nt paired-end sequencing.

### 2.4 Stereo-seq sample preparation and sequencing

Samples were embedded in Tissue-Tek OCT within 30 minutes after resected and wiped extra fluid. After frozen in dry ice, embedded samples were transferred to a -80°C freezer for storage until used. In a Dakewe CT520 cryostat, sample sections with 10μm were attached to Stereo-seq chips immediately.

The spatial transcriptomics data were obtained according to the protocol of STOmics Gene Expression Set-S1 on the website (https://www.stomics.tech/), which is an improved version of initial procedures. Briefly, 10 μm of tissue sections were adhered to the Stereo-seq chip and then heated for 3 minutes at 37 °C on a slide warmer. After incubation in methanol at -20 °C for 30 min, the chip was stained with nucleic acid dye (Thermo Fisher, Q10212) and imaged with a GO Optical Eclipse microscope. The cDNA products were released and purified from Stereo-seq chips after permeabilization for 6–24 min (optimized based on pre-testing for each sample), followed by reverse transcription. Indexed cDNA libraries were constructed according to the manufacturer’s protocol. Finally, cDNA libraries were sequenced on an MGI DNBSEQ-T7 sequencer (50 bp for read 1, 100 bp for read 2).

### 2.5 Quantification and statistical analysis

#### 2.5.1 snRNA-seq data processing

The gene expression matrix was generated using dnbc4tools software (v2.1.3). The UMI count matrix was converted into a Seurat object using the Seurat R package (v4.3.0) (25). Quality control criteria were applied as follows: low-quality nuclei with UMI counts <1200, detected genes <500, or mitochondrial UMI counts >5% were excluded; genes detected in fewer than 10 nuclei were removed. Following quality control, matrices from different samples were merged using Seurat to facilitate cross-sample comparison in subsequent analyses. Specifically, the merged count matrices were log-normalized using the NormalizeData function and scaled using the ScaleData function. The top 2000 variable genes were identified using the FindVariableFeatures function, and principal components (PCs) were calculated using the RunPCA function based on these genes. Data integration was performed using the Harmony algorithm (26). Nearest neighbors were computed based on the top 30 PCs, and cell clusters were identified using the FindClusters function (resolution = 0.8). The clustering resolution (0.8) was selected after evaluating multiple resolutions (0.2–1.6) to achieve stable and biologically interpretable clusters based on canonical marker expression. Clustering results were visualized using t-SNE or UMAP plots. To determine cell types for each cluster, marker genes were detected using the FindMarkers function in the Seurat package, and cell types were annotated using the ScType tool (27).

#### 2.5.2 Differential gene expression analysis

Differentially expressed genes (DEGs) were identified using the FindMarkers and FindAllMarkers functions from the Seurat package, which employed a Wilcoxon rank sum test with Bonferroni correction for multiple testing adjustment. Genes were considered DEGs if they exhibited a log-normalized expression difference ≥0.5 and an adjusted p-value <0.05.

To control for pseudoreplication arising from treating individual nuclei as independent observations, pseudobulk differential expression analysis was additionally performed by aggregating UMI counts per animal and testing SAP versus control using DESeq2, treating each rat as one biological replicate per group. Multiple testing correction strategies were applied according to analytical context, including Bonferroni adjustment for single-cell differential expression analyses, Benjamini–Hochberg false discovery rate (FDR) correction for enrichment and spatial analyses, and DESeq2’s default FDR framework for pseudobulk comparisons.

#### 2.5.3 Pseudotime analysis

Pseudotime analysis, trajectory construction, and calculation of pseudotime-dependent gene expression were performed using Monocle2 software (v2.26.0) (28). Dimension reduction was conducted using the DDRTree algorithm (v0.1.5). Cell trajectories were visualized based on cell subtypes and states. The Basic Differential Analysis algorithm implemented in Monocle2 was used to identify pseudotime-dependent differentially expressed genes. Significantly changed genes were identified using the differentialGeneTest function in Monocle2 with a q-value threshold of <0.0001.

#### 2.5.4 Cell–cell communication

Following cell type annotation, cell-cell communication analysis was performed using the CellChat pipeline (v2.1.2) (29). First, CellChat objects were constructed from the Seurat object. Next, precompiled Protein-Protein Interactions (PPIs) based on the CellChatDB.mouse database were used as prior network information. Then, communication probabilities were calculated using the computeCommunProb function. After that, cell-cell communication relationships were inferred and the communication network was aggregated with default parameters. Further, visualization of interaction counts was performed to display the aggregated cell-cell communication network and signaling output from each cell cluster. Finally, interactions between distinct cell subpopulations via putative ligand-receptor pairs were visualized using the ggplot2 R package. Because a rat-specific curated ligand–receptor database is currently limited, CellChatDB.mouse was used as a close proxy after gene symbol harmonization, an approach commonly adopted in rat single-cell transcriptomic studies.

#### 2.5.5 Transcription factor regulatory network analysis

Transcription factor (TF) modules were identified using the SCENIC Python workflow (v0.12.0) (30) with default parameters (tool official website: http://scenic.aertslab.org). In SCENIC, “regulons” were defined as a transcription factor together with its motif-validated direct target genes, inferred from co-expression and filtered by cis-regulatory motif enrichment. Regulon activity was quantified using AUCell scores. To quantify cell-type specificity of regulon activity, we computed the Regulon Specificity Score (RSS). Activated TFs were detected from the AUC (Area Under the Curve) matrix, and differentially activated TFs were statistically selected using the limma R package (31). To identify cluster-specific regulons (especially in analyses with multiple cell types where some regulons may be shared across cell types), the RSS (32) was applied for quantitative evaluation.

#### 2.5.6 Functional enrichment analysis

To systematically characterize the functional categories of genes, Gene Ontology (GO) term and KEGG pathway enrichment analyses were performed using KOBAS 2.0 (33). The hypergeometric test combined with the Benjamini-Hochberg false discovery rate (FDR) correction was applied to identify significantly enriched terms, with a corrected p-value threshold of <0.05.

#### 2.5.7 Stereo-seq spatial transcriptomics data analysis

Raw reads mapping was performed using STAR software with the mRatBN7.2 genome (https://www.ncbi.nlm.nih.gov/datasets/genome/GCF_015227675.2/). A single base mismatch was allowed to correct sequencing and PCR errors. Mapped reads were counted and annotated using SAW (v8.1), an open-source pipeline developed by BGI. The GEF output files from the SAW pipeline were used as input for downstream data analysis with Stereopy software (v1.6.0) (34). Given that each stereo-chip contained millions of DNA Nanoballs (DNBs, diameter: 220 nm), adjacent 50×50 DNBs were merged into one bin50 spot (25μm resolution), which served as the fundamental unit for downstream analysis. For each chip, spots with total UMI (Unique Molecular Identifier) counts <500, detected genes <100, or mitochondrial-derived UMI counts >15% were classified as low-quality and removed. Genes detected in fewer than 3 spots were also excluded. Batch effect correction across different chips was conducted using the batches_integrate function of Stereopy, and spot clustering was performed with the Leiden algorithm. Visualization of the spatial transcriptomics data was completed using Scanpy software (35).

#### 2.5.8 Detection of spatially variable genes

Spatially variable genes (SVGs) were identified using SPARK-X with default parameters (FDR < 0.05).

For each Stereo-seq sample, significant SVGs were ranked by adjusted p-value, and the top SVGs were used for GO biological process enrichment analysis.

Spatial expression patterns of the top 20 SVGs per sample were visualized to illustrate spatially structured inflammatory, epithelial, and ECM-related programs.

#### 2.5.9 Neighborhood and co-occurrence analysis

Spatial neighborhood enrichment and co-occurrence analyses were performed using squidpy, computing interaction z-scores and co-occurrence probabilities across multiple spatial radii.

These analyses quantified spatial proximities among cell types and supported the identification of SAP-associated epithelial–stromal–immune interactions.

#### 2.5.10 Other statistical analysis

Statistical analyses were performed using R software (version 4.0). Differences in cell-type proportions between SAP and control groups were assessed using the propeller() function from the speckle package, which models compositional data at the sample level. Comparisons between SAP and control groups for ELISA, qPCR, and Western blot assays were performed using unpaired two-tailed Student’s t-tests. A p-value < 0.05 was considered statistically significant. Data are presented as mean ± SEM, with n indicating the number of biological replicates.

## 3 Results

### 3.1 SAP Induces significant changes in intestinal cellular composition and spatial distribution

**Figure 1:**
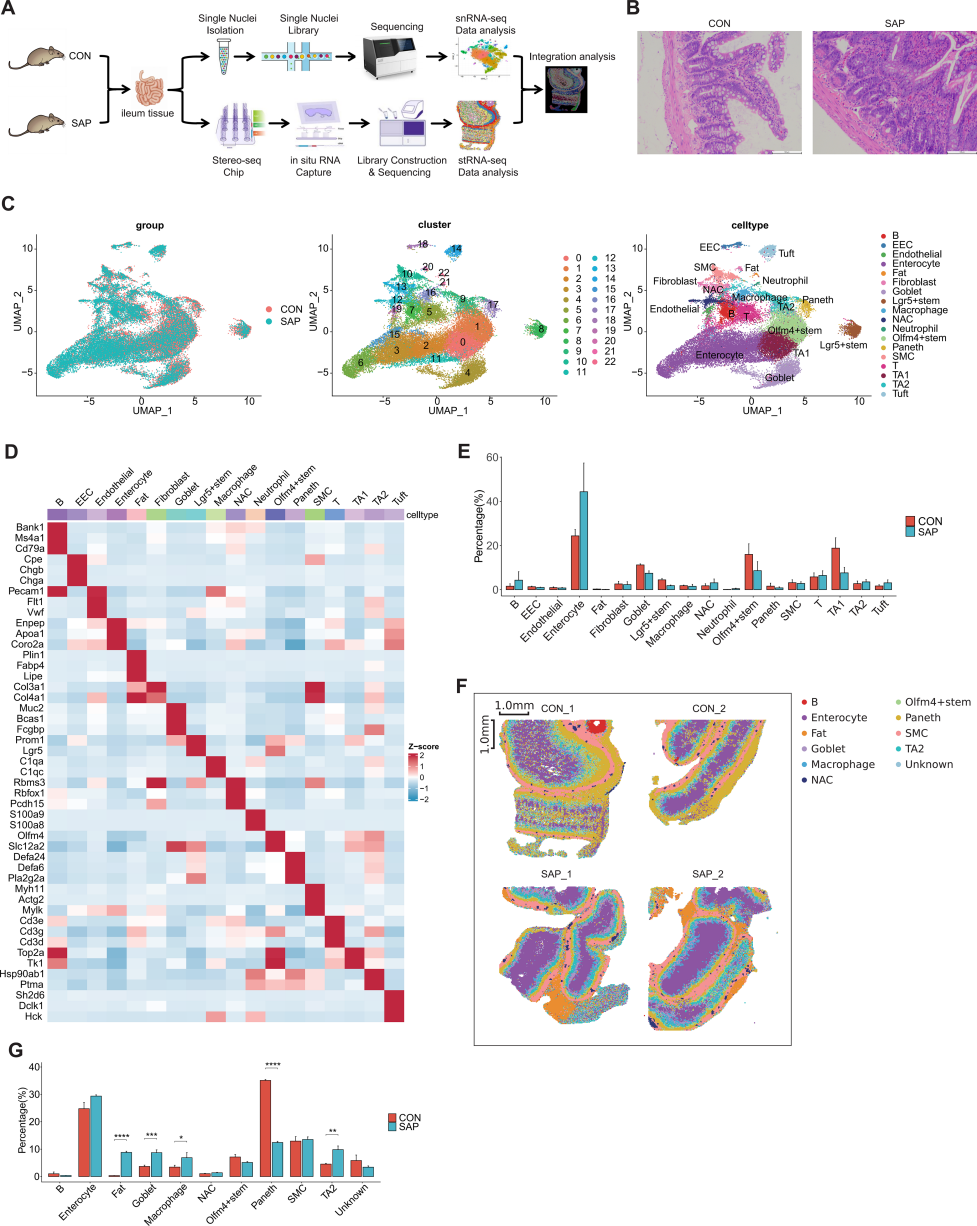
Single-nucleus transcriptome and spatial transcriptomics landscape of the ileal tissue of SAP and CON group rats. **(A)** Schematic illustration of the workflow for this study. **(B)** Representative Hematoxylin and Eosin (H&E)–stained ileal sections from CON and SAP rats. **(C)** UMAP plot of single-nucleus transcriptome profiles of SAP and CON group samples. Colors indicate groups, clusters and cell types. **(D)** Heatmap plot of marker genes for cell annotation. **(E)** Bar plot showing cell-type proportions (mean ± SEM) in snRNA-seq data. **(F)** Spatial transcriptomics profiles of SAP and CON group samples. Colors indicate cell types. **(G)** Bar plot showing cell-type proportions (mean ± SEM) in spatial transcriptomics (Stereo-seq) data. Statistical significance: ns, not significant; *P < 0.05; **P < 0.01; ***P < 0.001; ****P < 0.0001.

Histological examination of ileal tissues confirmed successful SAP model establishment. Control specimens displayed intact villus–crypt architecture with well-organized mucosal folds, whereas SAP samples exhibited villus shortening and collapse, epithelial necrosis and detachment, inflammatory infiltration, and vascular congestion (**Figure 1B**). Serum ELISA showed significantly elevated levels of amylase, TNF-α, and IL-6 in SAP rats compared with controls (unpaired two-tailed Student’s t-test; *P < 0.05; see **Supplementary Figure S1A** for symbol definitions). In addition, qPCR and Western blot analyses revealed increased ICAM-1 expression in SAP ileal tissues (unpaired two-tailed Student’s t-test; significance thresholds as defined in **Supplementary Figures S1B, C**) (**Supplementary Figures S1A–C**). Collectively, these findings confirmed successful SAP model establishment and supported the robustness of the model.

Single-nucleus RNA sequencing generated transcriptome data from 38, 999 high-quality nuclei (**Supplementary Table S1**). Distributions of key quality-control metrics (nCount_RNA, nFeature_RNA, and percent.mt) across all snRNA-seq samples are shown in **Supplementary Figures S2A–C**, supporting the filtering strategy described in the Methods. Unsupervised clustering identified 23 clusters, which were annotated into 18 cellular identities, including immune (B, T, macrophages, neutrophils), epithelial (enterocytes, enteroendocrine [EEC], goblet, Paneth, tuft), stem (Lgr5+, Olfm4+), transit-amplifying (TA1, TA2), and stromal populations (endothelial, adipocyte, fibroblast, neuron-associated cells [NAC], smooth muscle cells [SMC]) (**Figure 1C**, **Supplementary Figure S1D**). Marker-specific expression was confirmed by heatmap and bubble plot analyses (**Figure 1D**, **Supplementary Figure S1E**).

Quantitative analysis of snRNA-seq data revealed a trend toward decreased proportions of Lgr5^+^ and Olfm4^+^ stem cells, TA1 cells, goblet cells and Paneth cells in SAP, with a reciprocal increase in enterocytes (**Figure 1E**, **Supplementary Figure S1F**), although these differences were not statistically significant at the sample level.

Spatial transcriptomics generated 84, 115 bin50 spots after quality control (**Supplementary Table S2**). QC distributions for spatial transcriptomics (nCount_Spatial, nFeature_Spatial, and percent.mt) across all samples are likewise provided in **Supplementary Figures S2D–F**. Spatial transcriptomics was performed on two biological replicates per group. Annotation identified 10 major cell types: B cells, enterocytes, fat cells, goblet cells, macrophages, NAC, Olfm4+ stem cells, Paneth cells, SMC, and TA2, with one unannotated cluster named “unknown” (**Figure 1F**, **Supplementary Figure S1H**). Analysis of spatial cell-type composition showed a significant reduction in Paneth cells, accompanied by increases in fat cells, goblet cells, macrophages and TA2 cells in SAP ileum compared with controls, whereas enterocytes and Olfm4^+^ stem cells exhibited similar directional shifts but without statistically significant differences (**Figure 1G**, **Supplementary Figure S1G**).

Neighborhood enrichment analysis using permutation-derived z-scores revealed widespread SAP-associated changes in local spatial proximity across epithelial, immune, and stromal compartments (**Supplementary Figure S3**). Permutation-derived neighborhood enrichment z-scores were summarized at the sample level to compare spatial proximity patterns between groups (**Supplementary Figure S3**, **Supplementary Data sheet 1**). Positive z-scores indicate spatial co-localization, whereas negative values indicate spatial avoidance. Relative to controls, SAP samples showed higher neighborhood enrichment for the Olfm4^+^ stem–Paneth axis and Paneth–SMC pairs, with higher z-scores also observed for several Paneth-associated pairs, including Paneth–B and Paneth–unknown. In contrast, SAP samples exhibited lower neighborhood enrichment for multiple enterocyte-centered relationships, most prominently enterocyte–fat and enterocyte–TA2 pairs, as well as reduced enrichment for enterocyte–NAC, macrophage–enterocyte, fat–macrophage, and fat–SMC pairs (**Supplementary Figure S3**, **Supplementary Data sheet 1**).

### 3.2 SAP induces imbalance in intestinal stem cell and epithelial cell differentiation

**Figure 2:**
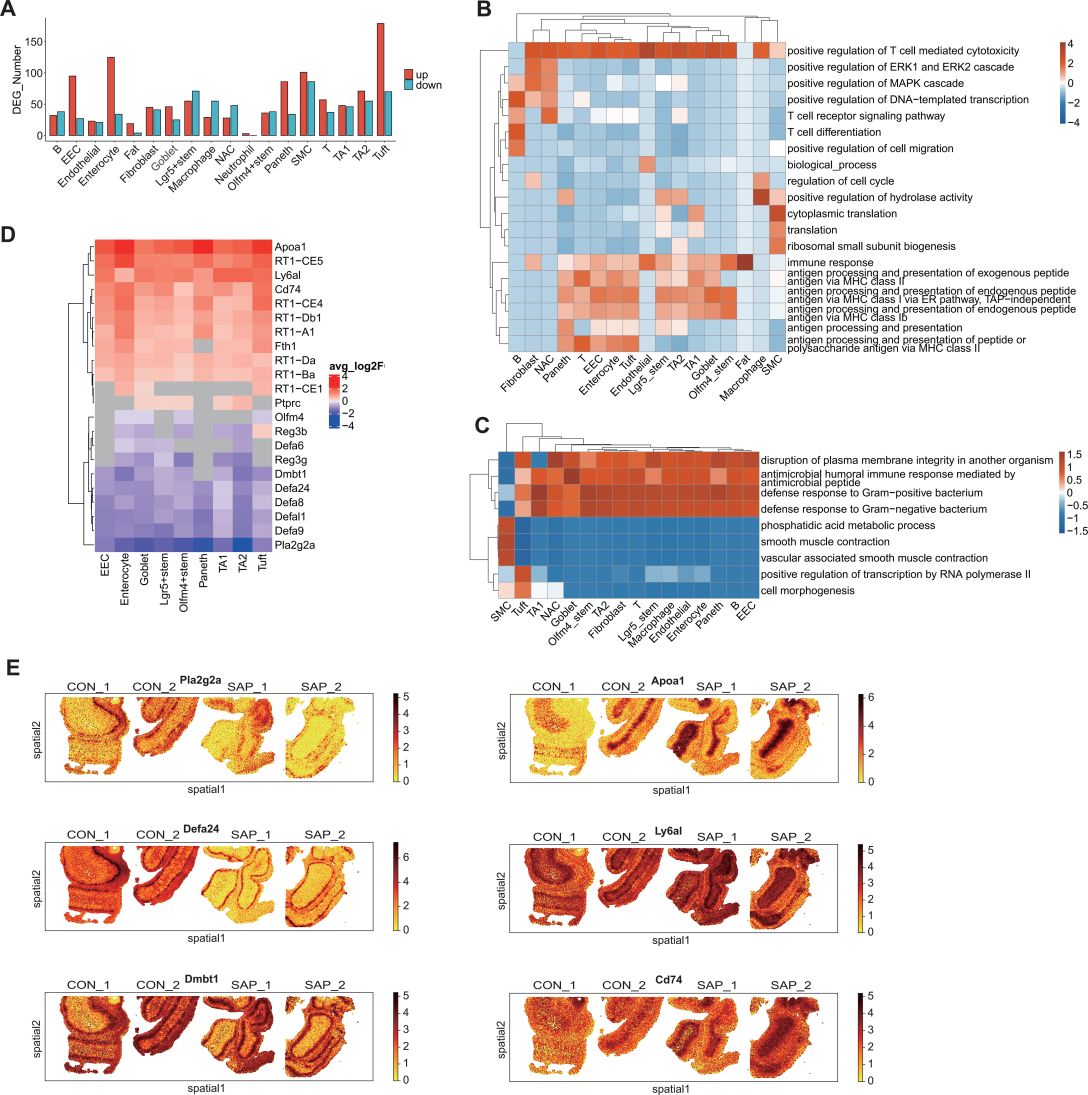
Analysis of DEGs in different cell populations from the ileal tissue of SAP and CON group samples. **(A)** Bar plot showing the number of upregulated and downregulated genes between the two groups in snRNA-seq data. **(B)** Gene Ontology (GO) enrichment analysis of biological processes of upregulated genes between SAP and CON. Top 3 terms were selected for each cell type and the heatmap shows enrichment q-values (scaled by column). **(C)** Gene Ontology (GO) enrichment analysis of biological processes of downregulated genes between SAP and CON. Top 3 terms were selected for each cell type and the heatmap shows enrichment q-values (scaled by column). **(D)** Heatmap plot showing the log2 fold-change value of genes related to immune response and antigen presentation in each cell type (SAP vs CON). **(E)** Spatial visualization of the expression levels of Pla2g2a, Defa24, Dmbt1, Ly6a1, Cd74 and Apoa1 genes in each spatial sample.

To characterize spatial patterns of mucosal gene activity, we identified spatially variable genes (SVGs) using SPARK-X (FDR < 0.05). GO biological process enrichment of the top-ranked SVGs (hypergeometric test with Benjamini–Hochberg FDR correction; FDR < 0.05) suggested a shift toward immune- and host defense–related programs in SAP, including responses to lipopolysaccharide and bacteria, although enrichment patterns varied across replicates (**Supplementary Figure S4**). Differential expression analysis across cell types identified extensive transcriptomic remodeling, with enterocytes, tuft, Paneth, and SMCs showing the largest DEG numbers (**Figure 2A**, **Supplementary Data sheets 2** and **3**). In non-immune epithelial subsets, up-regulated genes were enriched in antigen presentation and immune regulation pathways (e.g., Cd74, Apoa1), whereas down-regulated genes were enriched in antimicrobial defense responses (e.g., Defa24, Pla2g2a, Dmbt1) (**Figures 2B, C**, **Supplementary Figure S5**). Representative barrier/antimicrobial and antigen-presentation–related genes (Pla2g2a, Defa24, Dmbt1, Ly6a1, Cd74, and Apoa1) exhibited SAP-associated expression changes across epithelial lineages: Pla2g2a, Defa24, and Dmbt1 showed predominantly lower avg_log2FC values, whereas Ly6a1, Cd74, and Apoa1 showed higher avg_log2FC values in multiple epithelial cell types (**Figure 2D**). Spatial transcriptomics further visualized these differences at the tissue level (**Figure 2E**).

**Figure 3:**
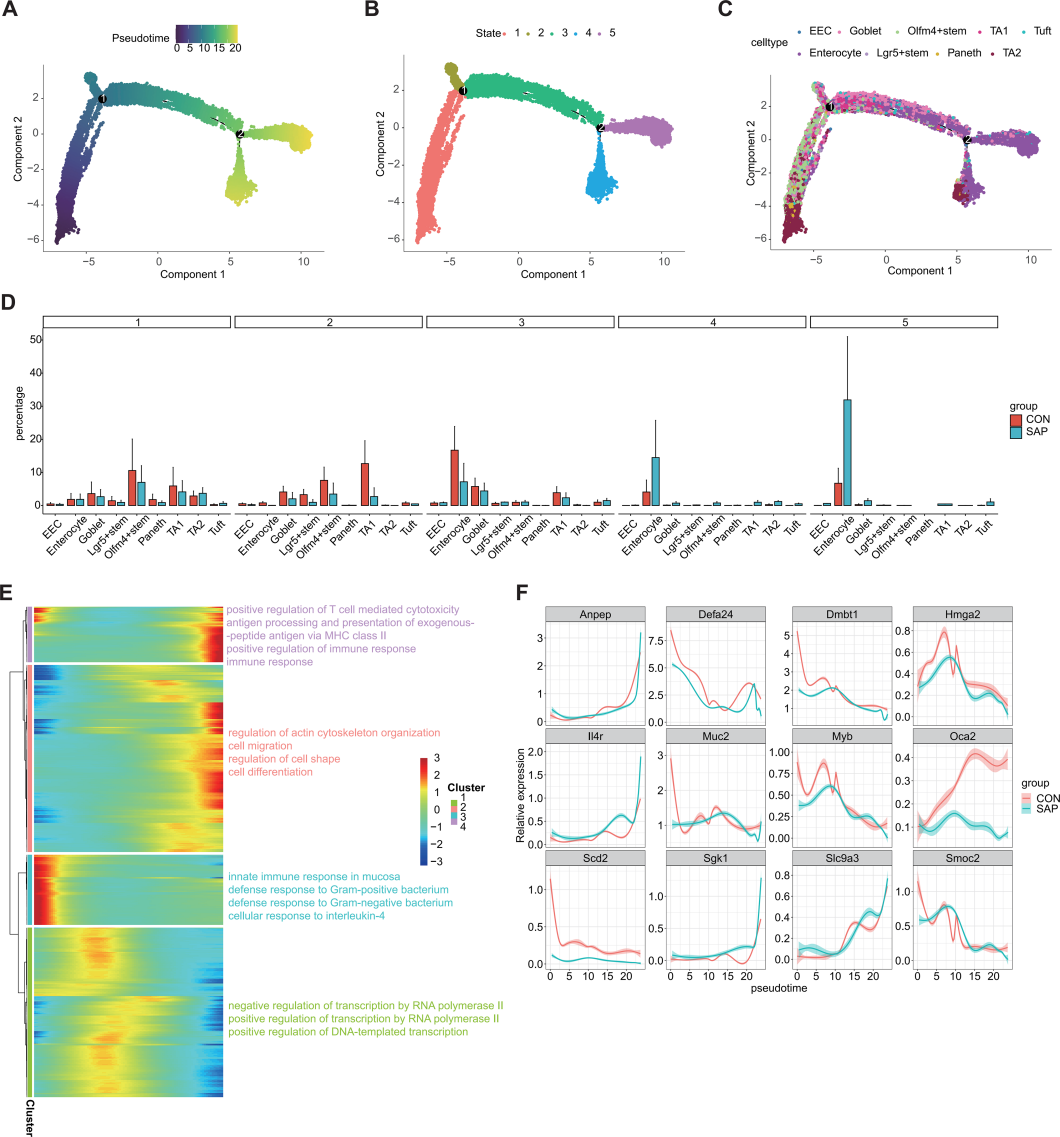
Pseudotime analysis of the epithelial cell subtypes of SAP and CON group samples in snRNA-seq data. **(A)** Pseudotime plots of the epithelial cell subtypes. Color-coded according to pseudotime value. **(B)** Pseudotime plots of the epithelial cell subtypes. Color-coded according to states. **(C)** Pseudotime plots of the epithelial cell subtypes. Color-coded according to cell types. **(D)** Bar plot showing the distribution of cells across states in each group. **(E)** Heatmap showing pseudotime-dependent genes; the right panel shows the most enriched GO biological process terms of these genes, grouped into 4 clusters using k-means. **(F)** Curve plot showing the expression level of genes in different groups along pseudotime.

Pseudotime trajectory analysis indicated a remodeled stem cell–epithelial differentiation continuum. Lgr5+ and Olfm4+ stem cells, together with TA1/TA2, were positioned at early pseudotime states, whereas enterocytes occupied terminal states (**Figures 3A–C**, **Supplementary Figure S6A**). SAP was associated with a relative depletion of early differentiation states (stem/TA-enriched) and an expansion of late enterocyte-dominant states (**Figure 3D**). Gene modules correlated with pseudotime progression were enriched in immune response, transcriptional regulation, cytoskeletal dynamics, and epithelial morphogenesis (**Figure 3E**). Genes from key pseudotime-associated modules (e.g., Anpep, Muc2, Smoc2, Hmga2, Myb, Defa24, Dmbt1) displayed distinct pseudotemporal patterns between groups (**Figure 3F**, **Supplementary Figure S6D**). Together, these data suggest that SAP shifts the epithelial differentiation landscape by depleting early stem/TA-enriched states and expanding terminally differentiated enterocyte states, contributing to epithelial imbalance. Consistent with the cell-level analysis, pseudobulk DESeq2 further supported down-regulation of stemness- and defense-related genes and up-regulation of inflammatory and ECM-remodeling programs in SAP, reinforcing the robustness and cross-platform consistency of these signatures.

### 3.3 SAP enhances intercellular communication with FN1 pathway mediating stem cell-epithelial cell interactions

**Figure 4:**
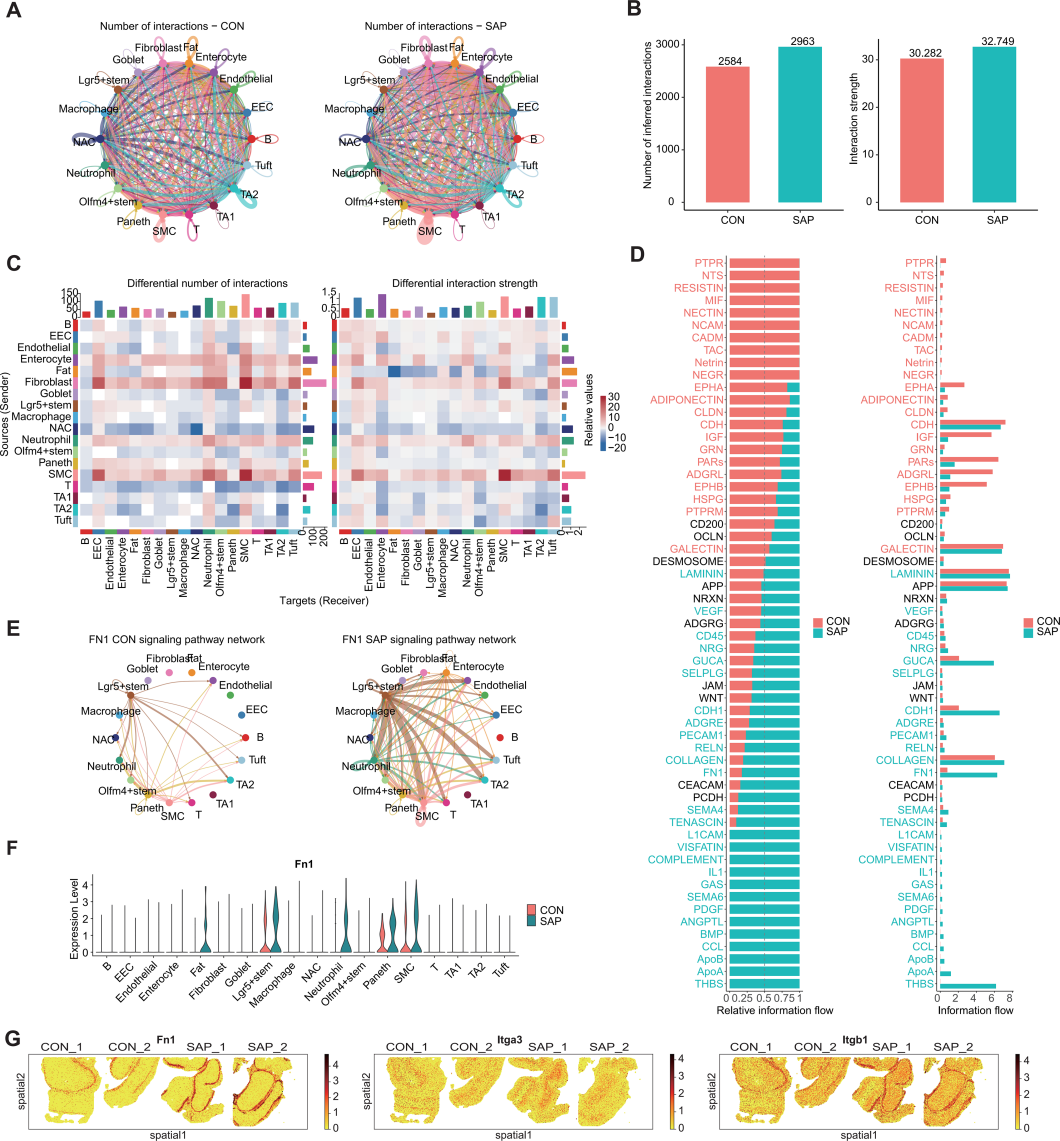
CellChat-based cell–cell communication analysis across intestinal cell types. **(A)** Network plot showing the total number of inferred ligand–receptor interactions among cell types in each group. Node size represents the total interaction counts per cell type; edge thickness represents the aggregated interaction strength (communication probability). **(B)** Bar plot showing the total number of inferred interactions (counts) and overall interaction strength (sum of communication probabilities) in each group. **(C)** Heatmap showing group differences in interaction counts and interaction strength across cell-type pairs. Values represent aggregated CellChat scores derived from inferred communication probabilities. **(D)** Bar plot showing signaling pathways with differences in overall information flow between groups. Overall information flow is defined as the summed communication probability of all ligand–receptor pairs within each pathway across all cell-type pairs. **(E)** FN1 signaling network split by group. Arrows indicate sender-to-receiver directionality; edge thickness reflects the inferred FN1 signaling strength between cell types. **(F)** Violin plot showing Fn1 expression levels across cell types in CON and SAP samples (normalized expression). **(G)** Spatial feature plots showing the spatial expression patterns of Fn1, Itga3, and Itgb1 in each Stereo-seq sample.

CellChat analysis revealed globally intensified intercellular communication in SAP, characterized by increased interaction diversity and strength compared with controls (**Figures 4A, B**). Enterocytes and SMCs showed the greatest gain in input–output signaling intensities (**Supplementary Figure S7A**), while SMCs displayed marked increases in output and enterocytes in input signals relative to controls (**Figure 4C**). Among signaling pathways, FN1 ranked highest by differential information flow between SAP and control groups (**Figure 4D**).

Network analysis predicted Lgr5+ stem cells as one of the major FN1 senders, with inferred signaling toward enterocytes, SMCs, and neutrophils (**Figure 4E**), and they consistently ranked among the top FN1 signal–emitting populations in both groups (**Supplementary Figure S7B**). In contrast, Paneth cells showed relatively weaker FN1 output in SAP (**Supplementary Figure S7B**), consistent with their reduced abundance observed in both snRNA-seq and spatial analyses (**Figures 1E, G**). Fn1, Itga3, and Itgb1 showed overall higher expression in SAP across stem and epithelial compartments, corroborated by spatial transcriptomic data (**Figures 4F, G**, **Supplementary Figure S7C**). These findings suggest that SAP-induced intestinal injury involves augmented ECM-mediated signaling, collectively outlining a stem cell–FN1–epithelial/SMC signaling axis in SAP.

### 3.4 Hmga2/Myb regulon activity is suppressed in intestinal stem cells during SAP and is associated with altered regenerative programs

**Figure 5:**
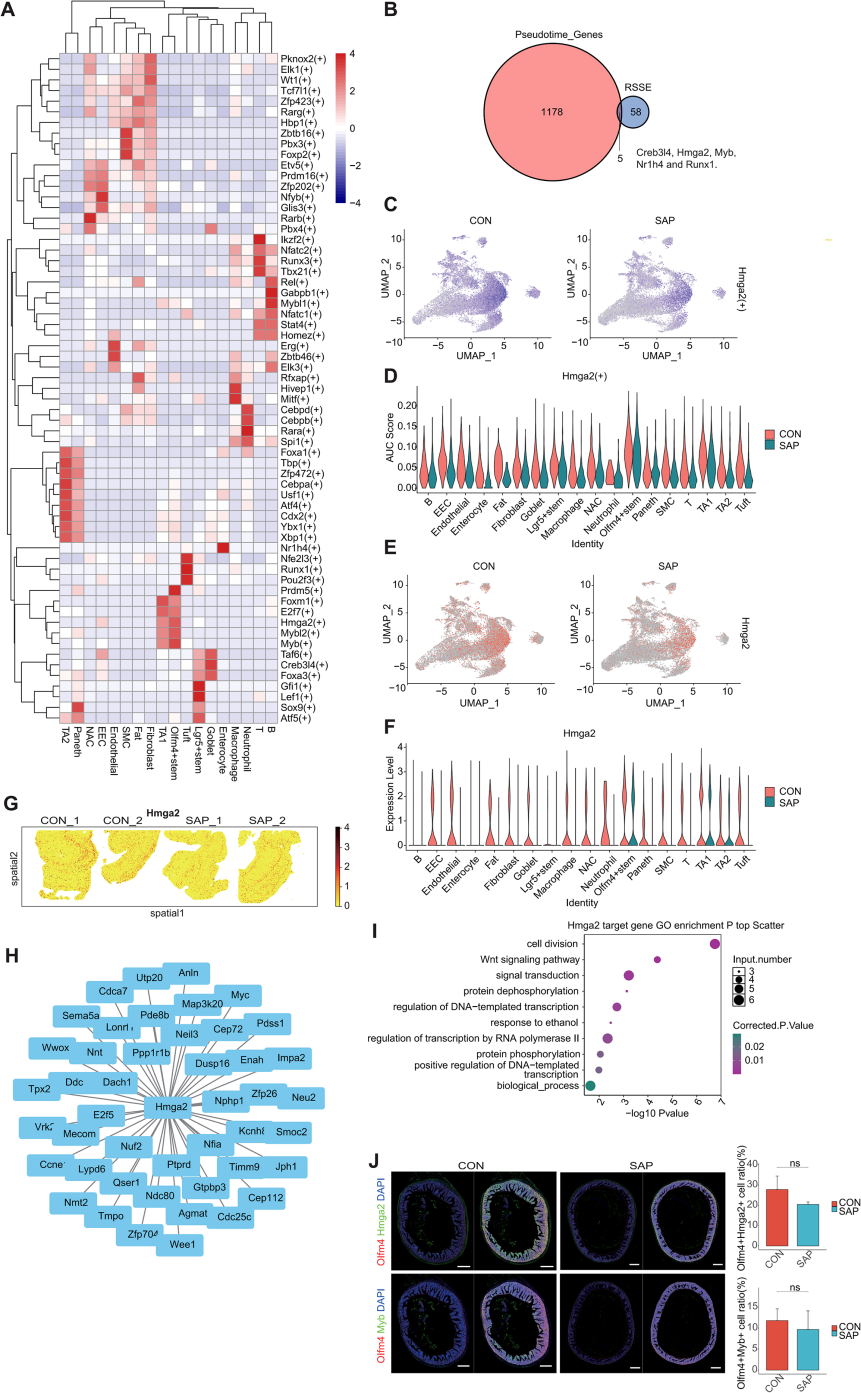
Transcription factor regulon analysis of different cell types. **(A)** Heatmap plot showing cell-type–specific transcription factor regulons in different cell types. Regulons were defined by the SCENIC workflow as a transcription factor and its motif-validated target genes. Cell-type specificity was quantified by the Regulon Specificity Score (RSS). Heatmap colors represent z-scored RSS values across cell types; higher values indicate stronger cell-type–specific regulon activity. **(B)** Venn diagram showing the overlap between the pseudotime-dependent genes and the cell type specific regulons. **(C)** UMAP plot depicting the activation level of transcription regulon Hmga2(+) in different groups. **(D)** Violin plots depict the activation level of transcription regulon Hmga2(+) of each cell type in different groups. **(E)** UMAP plot showing the expression levels of Hmga2 gene in different groups. **(F)** Violin plot showing the expression levels of Hmga2 gene of each cell type in different groups. **(G)** Spatial visualization of the expression levels of Hmga2 gene in each sample. **(H)** Network of transcription regulon Hmga2(+). **(I)** Bubble plot showing the most enriched GO biological process terms of the target genes of transcription factor Hmga2 predicted by pySCENIC. **(J)** Representative immunofluorescence staining showing reduced expression of Hmga2 and Myb in Olfm4^+^ stem cells. Scale bars, 500 μm. Quantification was performed on 6 fields of view per group, derived from 3 independent animals per group. Data are presented as mean ± SEM.

SCENIC analysis identified cell type–specific regulons, with Hmga2 and Myb emerging as prominent candidate regulators of stem cell fate (**Figures 5A, B**, **Supplementary Figure S8A**). Both regulon activity (AUC scores) and expression were reduced, particularly in SAP stem cell populations (**Figures 5C–F**, **Supplementary Figures S8B–E**). Spatial transcriptomics confirmed widespread downregulation of Hmga2/Myb expression (**Figure 5G**, **Supplementary Figure S8F**). Regulatory network reconstruction showed enrichment of target genes in cell cycle and proliferation pathways (**Figures 5H, I**, **Supplementary Figures S8G, H**).

Immunofluorescence with quantitative image analysis showed a decreasing trend in the proportions of Olfm4^+^ and Lgr5^+^ intestinal stem cells co-expressing Hmga2 or Myb in SAP compared with controls, although these differences did not reach statistical significance (**Figure 5J**, **Supplementary Figure S8I**). Collectively, these results implicate an Hmga2/Myb-associated regulon program that is attenuated in SAP, which may potentially contribute to reduced proliferative- and differentiation-associated regulatory activity in intestinal stem cells during injury.

## 4 Discussion

This study integrates single-nucleus RNA sequencing and Stereo-seq spatial transcriptomics to define how SAP reshapes the ileal mucosa at the levels of cellular composition, spatial organization, differentiation dynamics, and intercellular communication. Beyond recapitulating the concept that SAP disrupts epithelial homeostasis, our data add two key advances: first, they place epithelial and stromal remodeling into a spatial framework, and second, they nominate a coherent extracellular-matrix–centered signaling program—dominated by FN1—and a stem-cell–linked transcriptional program involving Hmga2/Myb as candidate regulators of impaired regeneration.

A major observation across modalities is that SAP skews the epithelial compartment toward a more enterocyte-dominant state while compromising the secretory/stem–progenitor axis. In the snRNA-seq data, Lgr5^+^/Olfm4^+^ stem cells, TA populations, goblet cells, and Paneth cells show consistent downward trends with a reciprocal increase in enterocytes, although most proportion changes do not reach statistical significance at the animal level, underscoring the variability expected in n=3 per group designs. Importantly, the spatial dataset independently supports a reduction in Paneth cells together with broader remodeling of epithelial programs. Functionally, epithelial subsets adopt an inflammatory/immune-interacting transcriptional phenotype, with increased expression of antigen presentation–associated genes (e.g., Cd74) and decreased expression of mucosal defense effectors (e.g., Defa family and other antimicrobial/barrier-related transcripts). Such “immune-like epithelialization” is consistent with the concept that intestinal barrier failure in SAP is accompanied by epithelial rewiring toward host defense and inflammatory signaling, a central theme in SAP–gut pathophysiology (36).

The spatial layer of our analysis clarifies that SAP is not simply a global shift in cell states, but a reorganization of local neighborhoods that likely correspond to disrupted crypt–villus microenvironments (37). Neighborhood enrichment analysis shows SAP-associated changes in the proximity structure among epithelial, immune, and stromal compartments, including strengthened local relationships involving the crypt niche (e.g., enrichment of Olfm4^+^ stem–Paneth and Paneth–SMC pairs) and weakened enrichment for several enterocyte-centered relationships. These patterns align with the idea that epithelial function is constrained by spatially defined cues—oxygen and nutrient gradients, extracellular matrix architecture, stromal scaffolding, and immune cell positioning—which collectively govern crypt maintenance and villus maturation. In this context, the spatially structured transcriptional programs identified by SPARK-X (and their enrichment in defense- and inflammation-related processes) provide quantitative support that SAP produces regionally organized injury programs rather than diffuse, spatially random changes.

Our pseudotime analysis further suggests that SAP remodels the epithelial differentiation continuum, with relative depletion of early stem/TA-enriched states and expansion of later enterocyte-dominant states. Biologically, this pattern is consistent with a stress-response mode in which progenitor reserves are consumed or suppressed while mature epithelial states expand, potentially reflecting compensatory differentiation and/or failure to sustain self-renewal. Notably, intestinal epithelial systems are known to retain bidirectional plasticity under stress, including dedifferentiation and reserve stem activation, but the balance of these processes is highly context- and niche-dependent (38). Here, the convergence of pseudotime shifts with spatial neighborhood remodeling supports a model in which SAP perturbs the crypt niche environment in ways that disfavour maintenance of early progenitor programs and bias the tissue toward terminal epithelial states.

A distinctive spatial finding in our dataset is the pronounced increase in “fat cell” signatures in SAP Stereo-seq samples. This observation is biologically plausible in the SAP setting and merits explicit interpretation. Visceral adipose depots—particularly mesenteric adipose tissue (MAT), which anatomically bridges the pancreas and intestine—are increasingly recognized as active participants in SAP severity and secondary intestinal injury. In rat SAP models, MAT inflammation correlates with disease severity and intestinal injury, and mechanistic work supports the concept that pancreatitis triggers strong inflammatory responses in mesenteric adipose sites in a lipase-dependent manner (39). More recently, MAT immune components have been implicated in intestinal damage during SAP, including MAT B lymphocytes promoting enteric injury through pyroptosis-related mechanisms (40). Therefore, the enrichment of fat-associated spatial signals in SAP may reflect a genuine expansion/activation of adipose-associated niches at the serosal/mesenteric interface rather than a trivial technical artifact. At the same time, we recognize that spatial capture can be sensitive to sampling depth and section orientation; we mitigated this by trimming attached mesenteric fat, avoiding visible Peyer’s patches, and applying the same dissection workflow across groups. In this framework, the spatial “fat cell” increase is best interpreted as a pathology-relevant feature of SAP that highlights the intestine–mesentery axis as a component of mucosal injury, and it provides a clear direction for follow-up: integrating histology/IF markers of adipocytes and MAT immune infiltration, and correlating adipose-proximal regions with epithelial inflammatory programs.

Intercellular communication analysis nominates FN1 signaling as a prominent pathway differentiating SAP from controls and places stem and stromal compartments into an ECM-centered network. FN1 is a key extracellular matrix component that signals through integrins to regulate adhesion, migration, survival, and proliferation, and integrin–FN1 coupling can engage canonical pathways such as FAK and PI3K/AKT in diverse contexts. In our CellChat results, Lgr5^+^ stem cells emerge as major predicted FN1 senders with inferred signaling to enterocytes and SMCs, supported by increased expression of Fn1 and integrin components in SAP across relevant compartments and corroborated by spatial expression patterns. While these data do not establish causality, they provide a coherent descriptive axis—stem cell–FN1–epithelial/SMC connectivity—consistent with a model in which ECM remodeling and altered adhesion/interaction landscapes accompany SAP injury (41, 42). Importantly, the neighborhood enrichment results add an orthogonal spatial layer by quantifying changes in local proximity among niche-associated populations, strengthening the interpretation that SAP modifies the physical/organizational substrate on which such ligand–receptor programs operate.

SCENIC analysis further implicates suppression of Hmga2/Myb regulon activity in stem cell populations in SAP. Hmga2 is a chromatin-associated architectural factor linked to stem cell self-renewal and regenerative capacity under stress, including classical evidence in somatic stem cell systems (43) and additional evidence that HMGA2-linked programs support self-renewal in stem/progenitor contexts. In parallel, Myb-associated transcriptional programs are tightly connected to proliferation and lineage regulation in progenitor contexts. In our data, reduced Hmga2/Myb activity aligns with decreased expression of proliferation/cell-cycle–related targets and with immunofluorescence evidence showing reduced proportions of Hmga2/Myb-positive stem cells in SAP (noting that some comparisons show trends that do not reach statistical significance). Together, these findings are consistent with the hypothesis that SAP suppresses a regenerative transcriptional module in the crypt stem compartment while the mucosa shifts toward terminal differentiation states.

The present study also informs how our SAP model complements and extends prior single-cell studies of AP-associated intestinal injury. Prior AP intestinal atlases have highlighted early immune triggers—most notably mast cell activation and mast cell–derived CCL5 as a driver of barrier dysfunction in early time windows (20). In our 24-hour SAP time point and snRNA-seq workflow, mast cells are not recovered as a major distinct cluster, suggesting that mast cell signals may be temporally restricted to earlier phases, below detection due to low abundance, or less efficiently captured by nuclear profiling relative to whole-cell approaches. By contrast, our dataset emphasizes later-stage epithelial–stromal remodeling, ECM/FN1-centric communication, and niche-associated transcriptional suppression in stem compartments, thereby complementing early-phase immune-trigger models and highlighting time-dependent mechanisms across the AP/SAP trajectory. In addition, intestinal fibroblasts—recognized as diverse and functionally important orchestrators of epithelial homeostasis, ECM organization, and repair responses (12)—appear as a defined stromal population in our snRNA-seq landscape. Although fibroblast-centered mechanistic claims are not the focus of our current results, the prominent ECM remodeling signals and the FN1/integrin axis naturally motivate deeper dissection of fibroblast–SMC–epithelial crosstalk in future studies, ideally with targeted spatial annotation of stromal subtypes and validation of ECM deposition and stiffness-associated programs.

Several limitations should be considered when interpreting these findings. The number of animals is modest (snRNA-seq n=3 per group; Stereo-seq n=2 per group), which is common in high-dimensional single-cell/spatial studies but constrains power for sample-level proportion testing and increases sensitivity to inter-animal variability. We mitigated this by applying stringent QC and integration, using sample-level compositional inference (propeller) and pseudobulk DESeq2 to reduce pseudoreplication, and validating key biological features with orthogonal assays (histology, ELISA, qPCR/Western blot, and immunofluorescence). Second, snRNA-seq may underrepresent cytoplasmic transcripts and introduces modality-specific biases compared with whole-cell approaches. Third, pseudotime, ligand–receptor inference, and regulon activity are correlative; functional perturbation of FN1/integrin signaling or restoration of Hmga2/Myb programs will be required to establish causality. Finally, the sodium taurocholate-induced rat model captures core features of SAP but cannot fully recapitulate the heterogeneity of human disease. Because ileal biopsies from unstable SAP patients are rarely feasible outside limited clinical contexts, direct human single-cell/spatial validation remains challenging. Accordingly, this work should be viewed as mechanistic and hypothesis-generating, providing a spatially informed cellular framework. Future directions include ethically feasible acquisition of human intestinal materials, patient-derived ileal organoids, and spatially explicit trajectory inference methods to map differentiation states back onto crypt–villus regions and to test whether the stem-cell depletion, FN1-centered communication, and Hmga2/Myb suppression identified here generalize to human SAP.

## 5 Conclusion

In summary, by integrating single-nucleus and spatial transcriptomics, this study constructs a multi-dimensional atlas of SAP-associated ileal injury. We identify a consistent shift of epithelial programs toward immune interaction and reduced mucosal defense, accompanied by a redistribution of epithelial states along a stem/TA-to-enterocyte continuum and by quantitative remodeling of spatial neighborhood architecture. Spatial profiling further highlights significant depletion of Paneth cells and enrichment of fat-cell–associated signals, pointing to a potential intestine–mesentery axis in SAP. At the intercellular communication level, FN1-ranked ECM signaling emerges as a prominent pathway differentiating SAP from controls, while SCENIC nominates attenuated Hmga2/Myb regulon activity as a stem-cell–linked transcriptional feature associated with impaired regenerative programs. Together, these findings refine the spatial and cellular understanding of intestinal barrier failure in SAP and provide testable hypotheses implicating ECM/FN1 signaling and Hmga2/Myb-associated regulatory programs as candidate targets for future barrier-preserving strategies.

## Data Availability

The data presented in the study are deposited in the GEO repository, accession number GSE317063.

## Supplementary material

Supplementary Figure 1 Single-nucleus transcriptome and spatial transcriptomics landscape of the ileal tissue of SAP and CON group rats. **(A)** Serum levels of TNF-α, amylase, and IL-6 measured by ELISA (n = 3 per group). Bars indicate mean ± SEM; dots represent individual animals. Unpaired two-tailed Student’s t-test. **(B)** Relative Icam1 mRNA expression quantified by qPCR (n = 3 per group). Values were normalized to Gapdh and calculated using the 2^−ΔΔCt method. Bars indicate mean ± SEM; dots represent individual animals. Unpaired two-tailed Student’s t-test. **(C)** Western blot analysis of ICAM1 protein in ileal tissues with densitometric quantification (n = 3 per group). Bars indicate mean ± SEM; dots represent individual animals. Unpaired two-tailed Student’s t-test. **(D)** t-SNE visualization of integrated snRNA-seq nuclei from CON and SAP ileum, colored by experimental group, cluster, and annotated cell type. **(E)** Dot plot showing canonical marker gene expression used for cell-type annotation across snRNA-seq clusters. **(F)** Stacked bar plot showing cell-type proportions for each individual snRNA-seq sample. **(G)** Stacked bar plot showing cell-type proportions for each individual Stereo-seq sample. **(H)** H&E-stained ileal sections corresponding to the Stereo-seq samples (CON_1, CON_2, SAP_1, SAP_2), providing matched histological context for spatial transcriptomic profiling. Statistical significance: ns, not significant; *P < 0.05; **P < 0.01; ***P < 0.001; ****P < 0.0001.

Supplementary Figure 2 QC of snRNA-seq and spatial transcriptomics (Stereo-seq) data. **(A)** Violin plots showing the distribution of nCount_RNA in different samples of snRNA-seq data. **(B)** Violin plots showing the distribution of nFeature_RNA in different samples of snRNA-seq data. **(C)** Violin plots showing the distribution of percent.mt in different samples of snRNA-seq data. **(D)** Violin plots showing the distribution of nCount_Spatial in different samples of Stereo-seq data. **(E)** Violin plots showing the distribution of nFeature_Spatial in different samples of Stereo-seq data. **(F)** Violin plots showing the distribution of percent.mt in different samples of Stereo-seq data.

Supplementary Figure 3 Spatial neighborhood analysis of Stereo-seq data. **(A)** Heatmap showing the neighborhood enrichment z-score for each cell–cell interaction.

Supplementary Figure 4 Spatially variable genes (SVGs) analysis of Stereo-seq data. **(A)** Spatial visualization of the expression levels of representative SVGs in the four Stereo-seq samples. **(B)** Bubble plot showing the most enriched GO biological process terms of the top 50 SVGs in each Stereo-seq sample.

Supplementary Figure 5 Analysis of DEGs in different cell populations from the ileal tissue of SAP and CON group samples. **(A)** Heatmap showing the most enriched KEGG pathways of upregulated genes between SAP and CON groups in snRNA-seq data. **(B)** Heatmap showing the most enriched KEGG pathways of downregulated genes between SAP and CON groups in snRNA-seq data.

Supplementary Figure 6 Pseudotime analysis of the epithelial cell subtypes of SAP and CON group samples in snRNA-seq data. **(A)** The minimum spanning tree of all epithelial cells, color-coded according to cell types. **(B)** Density plot showing the distribution of pseudotime values of the epithelial cells. Color-coded according to states. **(C)** Bar plot showing the distribution of cells across states in each group (mean ± SEM). **(D)** Scatter plot showing gene normalized expression levels across the pseudotime trajectory. Cells are colored by states.

Supplementary Figure 7 Communication analysis of different cell types. **(A)** Bubble plot showing the incoming and outgoing communication interaction strength of all the cell types in different groups. **(B)** Heatmap showing the differential strength of interactions for the FN1 signaling pathway between groups. **(C)** Violin plot showing the expression levels of Itga3 and Itgb1 in each cell type of SAP and CON group samples.

Supplementary Figure 8 Transcription factor regulon analysis of different cell types. **(A)** Dot plot showing specific transcription regulons in different cell types. **(B)** UMAP plot depicting the activation level of transcription regulon Myb(+) in different groups. **(C)** Violin plots depicting the activation level of transcription regulon Myb(+) of each cell type in different groups. **(D)** UMAP plot showing the expression levels of Myb in different groups. **(E)** Violin plot showing the expression levels of Myb in each cell type in different groups. **(F)** Spatial visualization of the expression levels of Myb in each sample. **(G)** Network of transcription regulon Myb(+). **(H)** Bubble plot showing the most enriched KEGG pathways of the target genes of transcription factor Hmga2 predicted by pySCENIC. **(I)** Representative immunofluorescence staining showing reduced expression of Hmga2 and Myb in Lgr5^+^ stem cells. Scale bars, 500 μm. Quantification was performed on 6 fields of view per group, derived from 3 independent animals per group. Data are presented as mean ± SEM.

Supplementary Table 1 Summary of snRNA-seq data quality metrics for ileal tissues from CON and SAP rats.

Supplementary Table 2 Summary of Stereo-seq spatial transcriptomics data quality metrics for ileal tissues from CON and SAP rats.

SUPPLEMENTARY DATA SHEET 1 Neighborhood enrichment z-scores for spatial co-localization among cell types in Stereo-seq data (CON_1, CON_2, SAP_1, SAP_2). Positive z-scores indicate spatial enrichment (co-localization), whereas negative z-scores indicate spatial avoidance (depletion).

SUPPLEMENTARY DATA SHEET 2 Number of differentially expressed genes (DEGs) in each cell type between CON and SAP groups.

SUPPLEMENTARY DATA SHEET 3 List of differentially expressed genes (DEGs) in each cell type between CON and SAP groups.
