# Supplementary figures and images for: Single-nucleus and spatial transcriptomics reveal intestinal cellular heterogeneity, differentiation, and cell communication mechanisms in SAP-induced intestinal injury
Source: Front Immunol. 2026 Jan 30;17:1719902. doi: 10.3389/fimmu.2026.1719902 (PMC12901460; doi:10.3389/fimmu.2026.1719902)

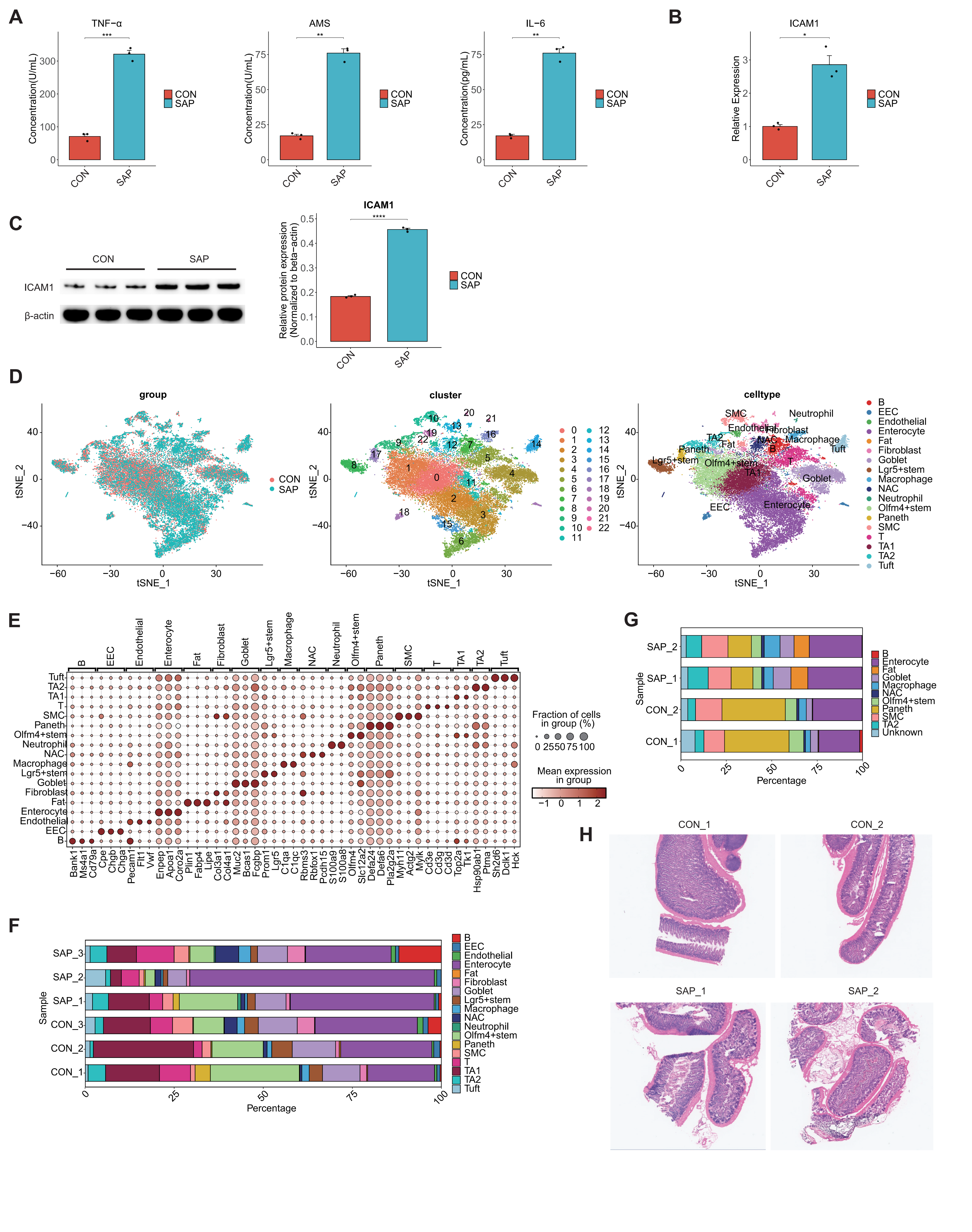

Supplement: Supplementary Figure 1 — Single-nucleus transcriptome and spatial transcriptomics landscape of the ileal tissue of SAP and CON group rats. (A) Serum levels of TNF-α, amylase, and IL-6 measured by ELISA (n = 3 per group). Bars indicate mean ± SEM; dots represent individual animals. Unpaired two-tailed Student’s t-test. (B) Relative Icam1 mRNA expression quantified by qPCR (n = 3 per group). Values were normalized to Gapdh and calculated using the 2^−ΔΔCt method. Bars indicate mean ± SEM; dots represent individual animals. Unpaired two-tailed Student’s t-test. (C) Western blot analysis of ICAM1 protein in ileal tissues with densitometric quantification (n = 3 per group). Bars indicate mean ± SEM; dots represent individual animals. Unpaired two-tailed Student’s t-test. (D) t-SNE visualization of integrated snRNA-seq nuclei from CON and SAP ileum, colored by experimental group, cluster, and annotated cell type. (E) Dot plot showing canonical marker gene expression used for cell-type annotation across snRNA-seq clusters. (F) Stacked bar plot showing cell-type proportions for each individual snRNA-seq sample. (G) Stacked bar plot showing cell-type proportions for each individual Stereo-seq sample. (H) H&E-stained ileal sections corresponding to the Stereo-seq samples (CON_1, CON_2, SAP_1, SAP_2), providing matched histological context for spatial transcriptomic profiling. Statistical significance: ns, not significant; *P < 0.05; **P < 0.01; ***P < 0.001; ****P < 0.0001. [file Image1.tif]

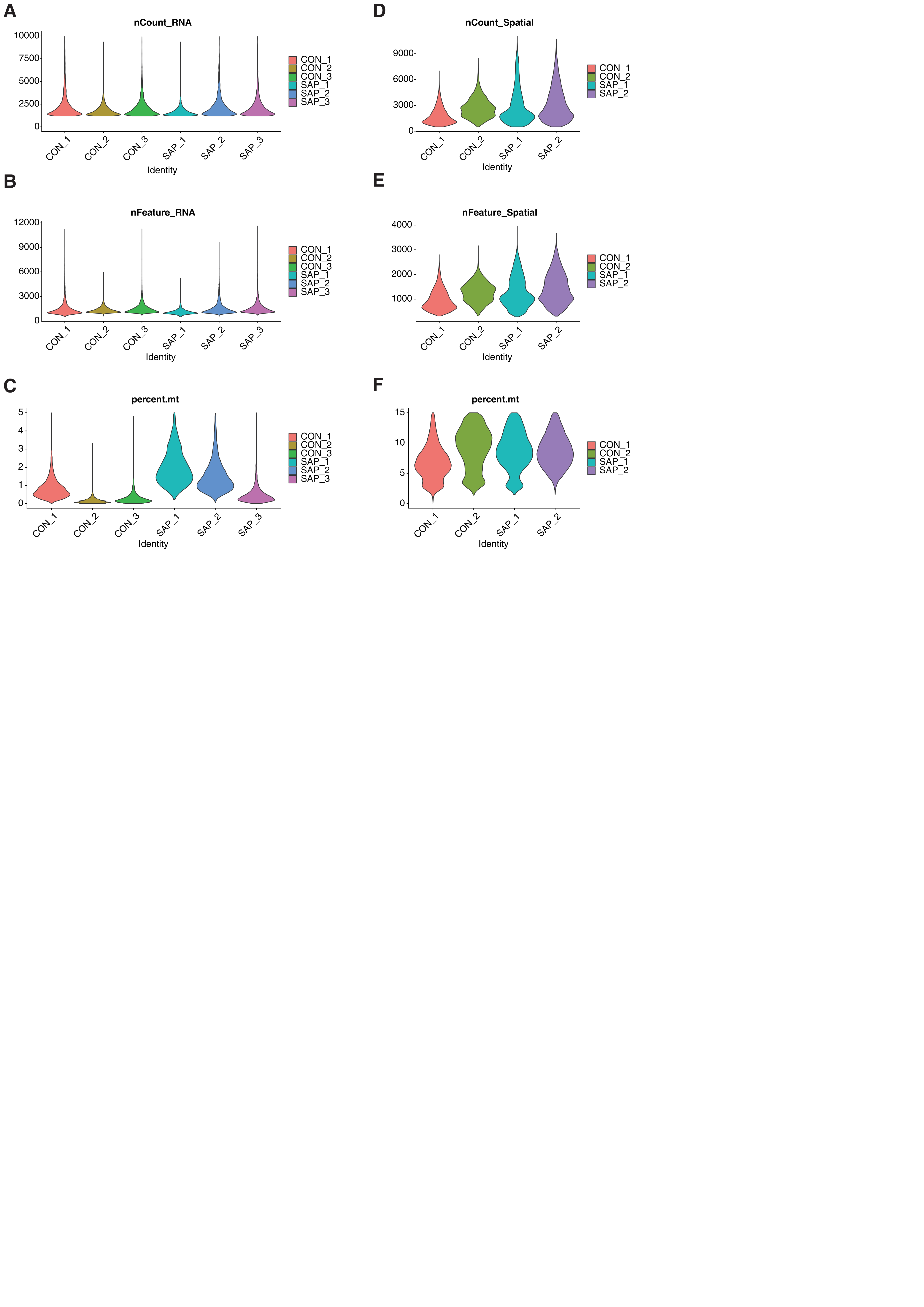

Supplement: Supplementary Figure 2 — QC of snRNA-seq and spatial transcriptomics (Stereo-seq) data. (A) Violin plots showing the distribution of nCount_RNA in different samples of snRNA-seq data. (B) Violin plots showing the distribution of nFeature_RNA in different samples of snRNA-seq data. (C) Violin plots showing the distribution of percent.mt in different samples of snRNA-seq data. (D) Violin plots showing the distribution of nCount_Spatial in different samples of Stereo-seq data. (E) Violin plots showing the distribution of nFeature_Spatial in different samples of Stereo-seq data. (F) Violin plots showing the distribution of percent.mt in different samples of Stereo-seq data. [file Image2.tif]

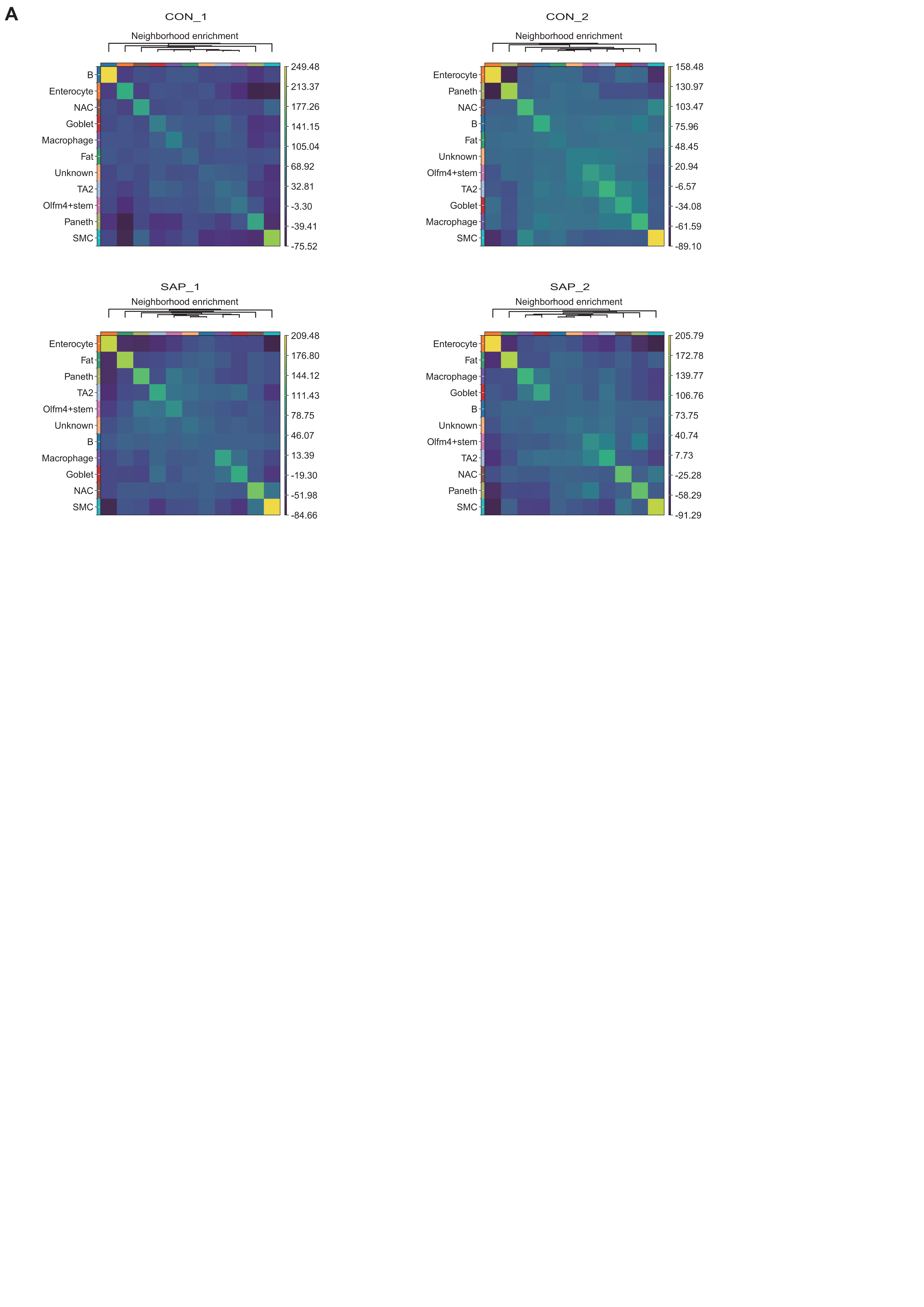

Supplement: Supplementary Figure 3 — Spatial neighborhood analysis of Stereo-seq data. (A) Heatmap showing the neighborhood enrichment z-score for each cell–cell interaction. [file Image3.tif]

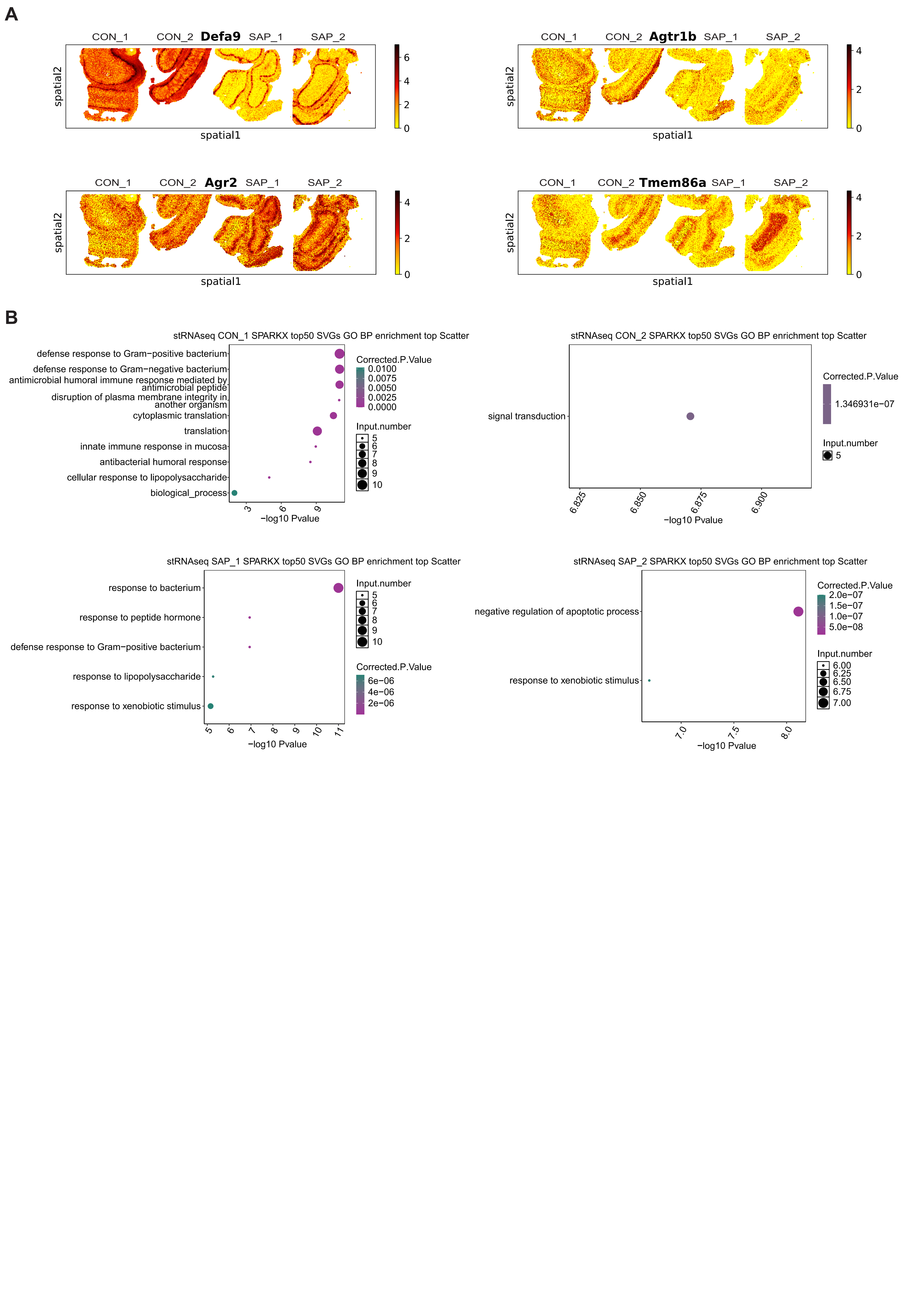

Supplement: Supplementary Figure 4 — Spatially variable genes (SVGs) analysis of Stereo-seq data. (A) Spatial visualization of the expression levels of representative SVGs in the four Stereo-seq samples. (B) Bubble plot showing the most enriched GO biological process terms of the top 50 SVGs in each Stereo-seq sample. [file Image4.tif]

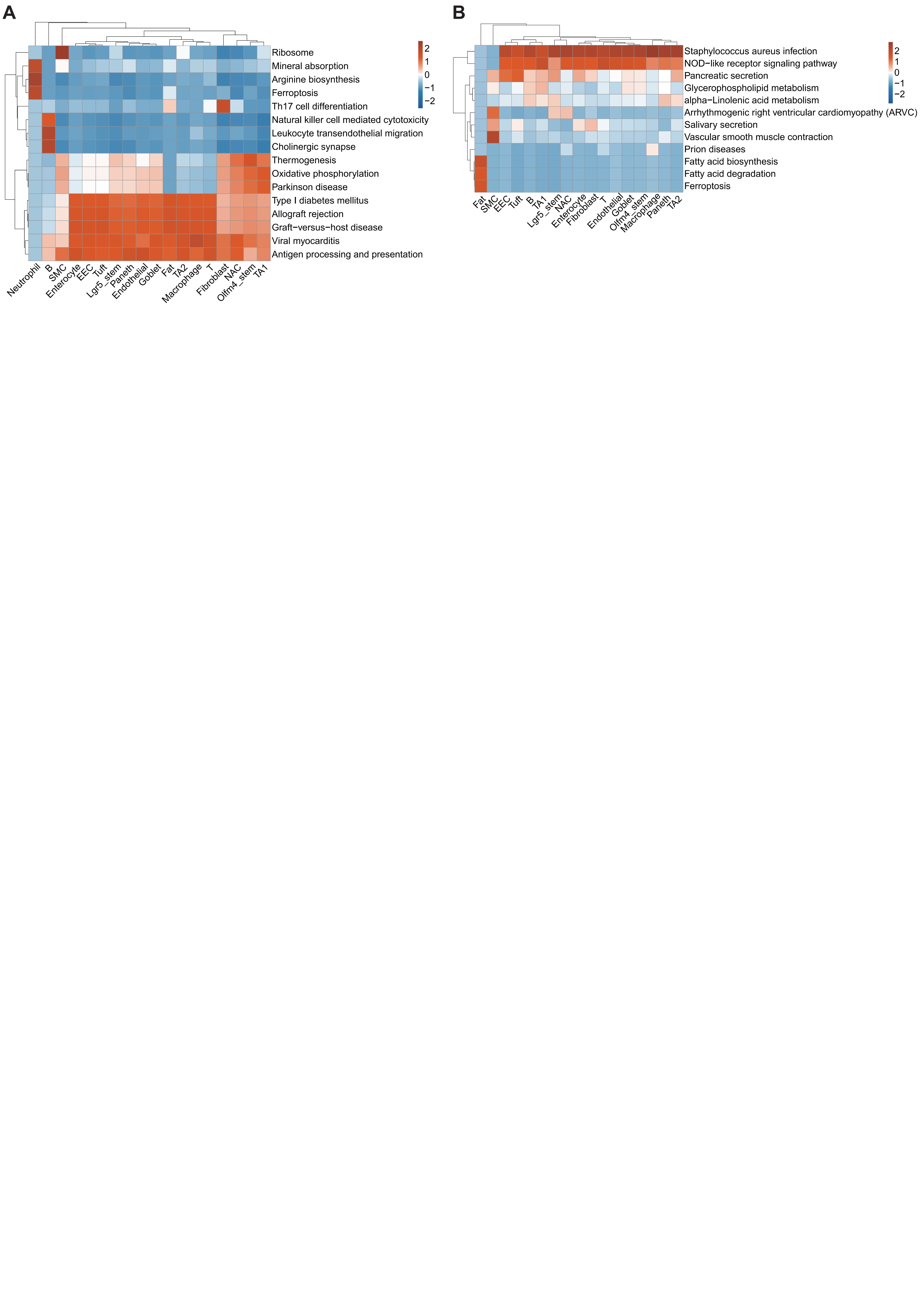

Supplement: Supplementary Figure 5 — Analysis of DEGs in different cell populations from the ileal tissue of SAP and CON group samples. (A) Heatmap showing the most enriched KEGG pathways of upregulated genes between SAP and CON groups in snRNA-seq data. (B) Heatmap showing the most enriched KEGG pathways of downregulated genes between SAP and CON groups in snRNA-seq data. [file Image5.tif]

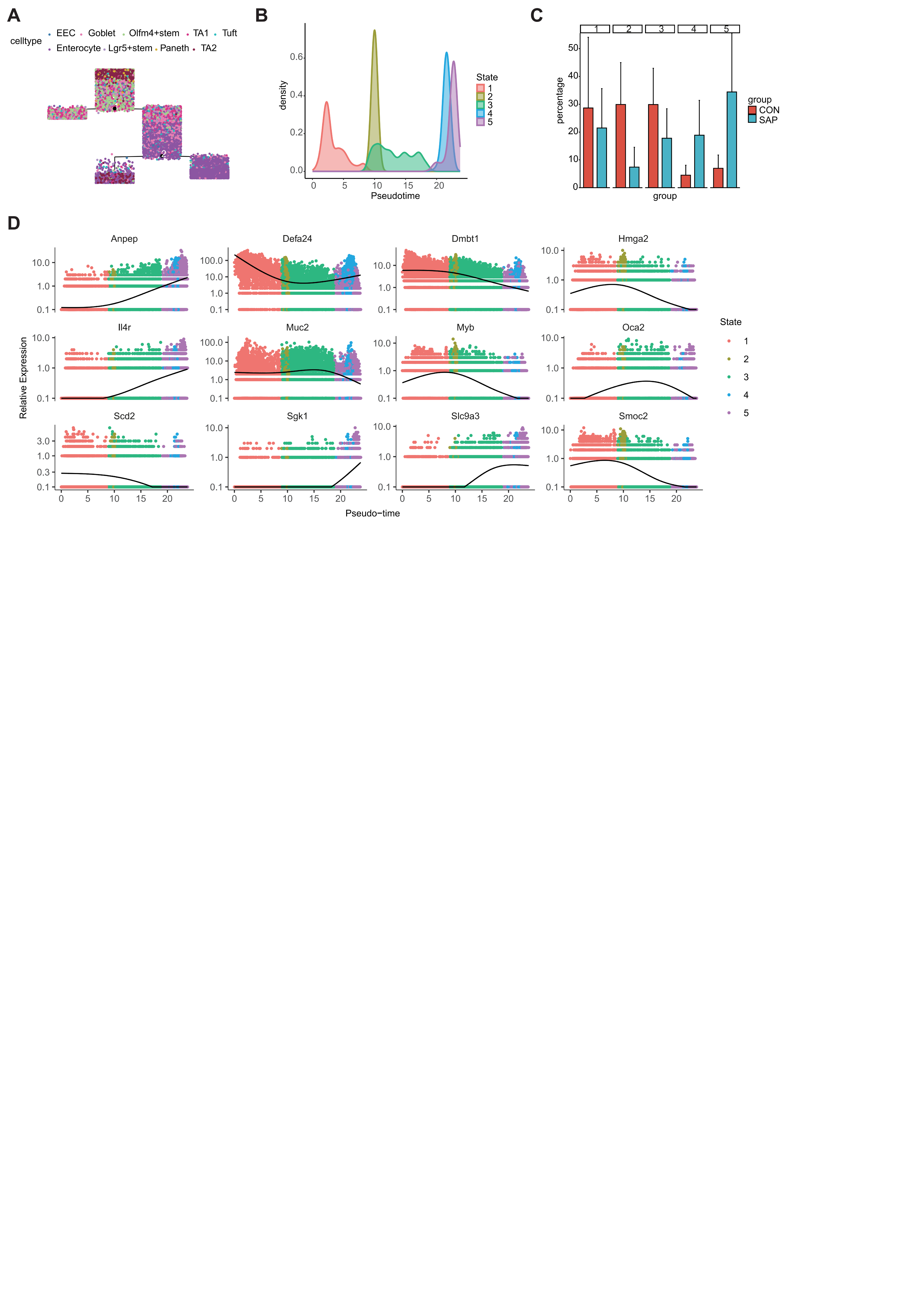

Supplement: Supplementary Figure 6 — Pseudotime analysis of the epithelial cell subtypes of SAP and CON group samples in snRNA-seq data. (A) The minimum spanning tree of all epithelial cells, color-coded according to cell types. (B) Density plot showing the distribution of pseudotime values of the epithelial cells. Color-coded according to states. (C) Bar plot showing the distribution of cells across states in each group (mean ± SEM). (D) Scatter plot showing gene normalized expression levels across the pseudotime trajectory. Cells are colored by states. [file Image6.tif]

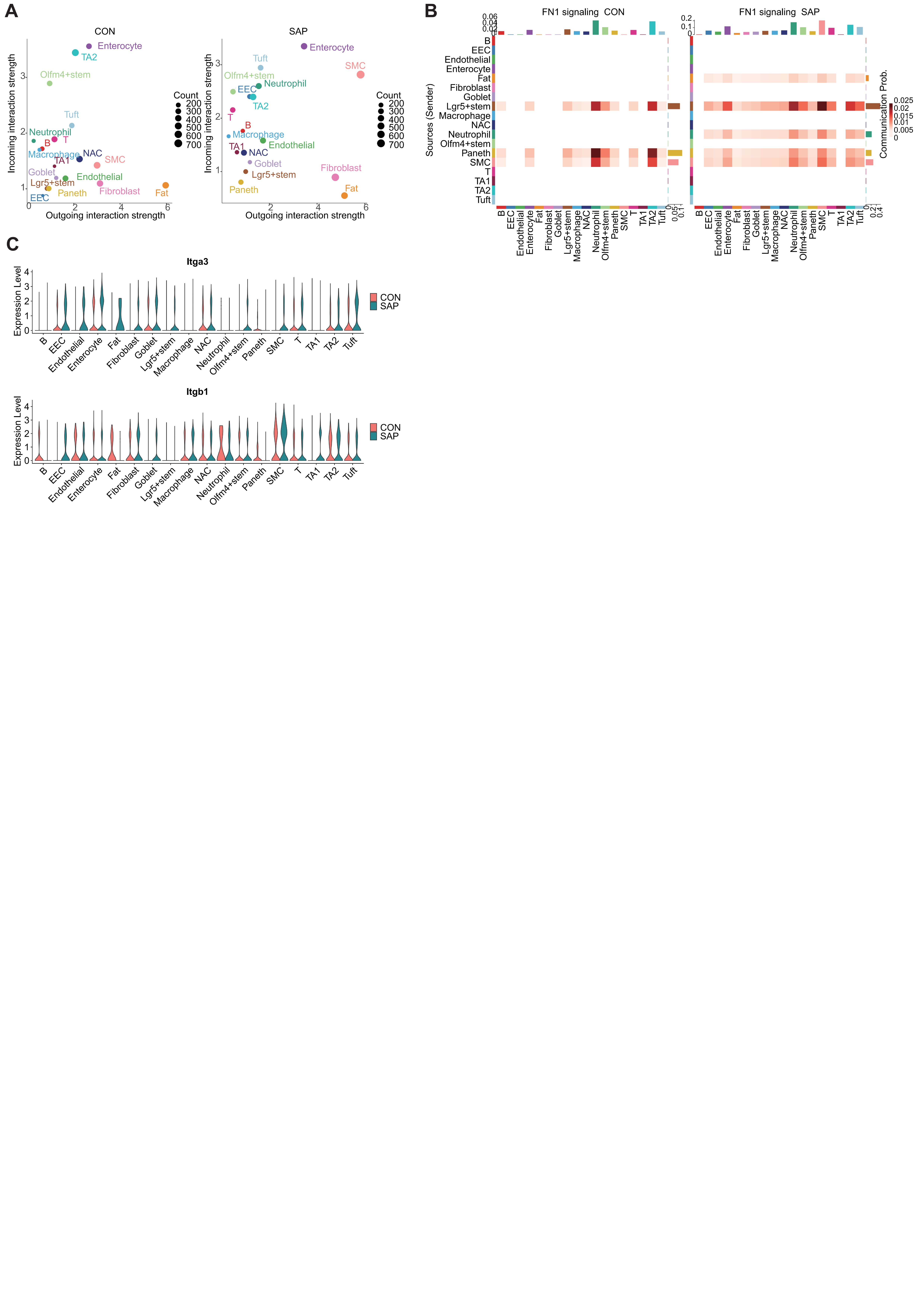

Supplement: Supplementary Figure 7 — Communication analysis of different cell types. (A) Bubble plot showing the incoming and outgoing communication interaction strength of all the cell types in different groups. (B) Heatmap showing the differential strength of interactions for the FN1 signaling pathway between groups. (C) Violin plot showing the expression levels of Itga3 and Itgb1 in each cell type of SAP and CON group samples. [file Image7.tif]

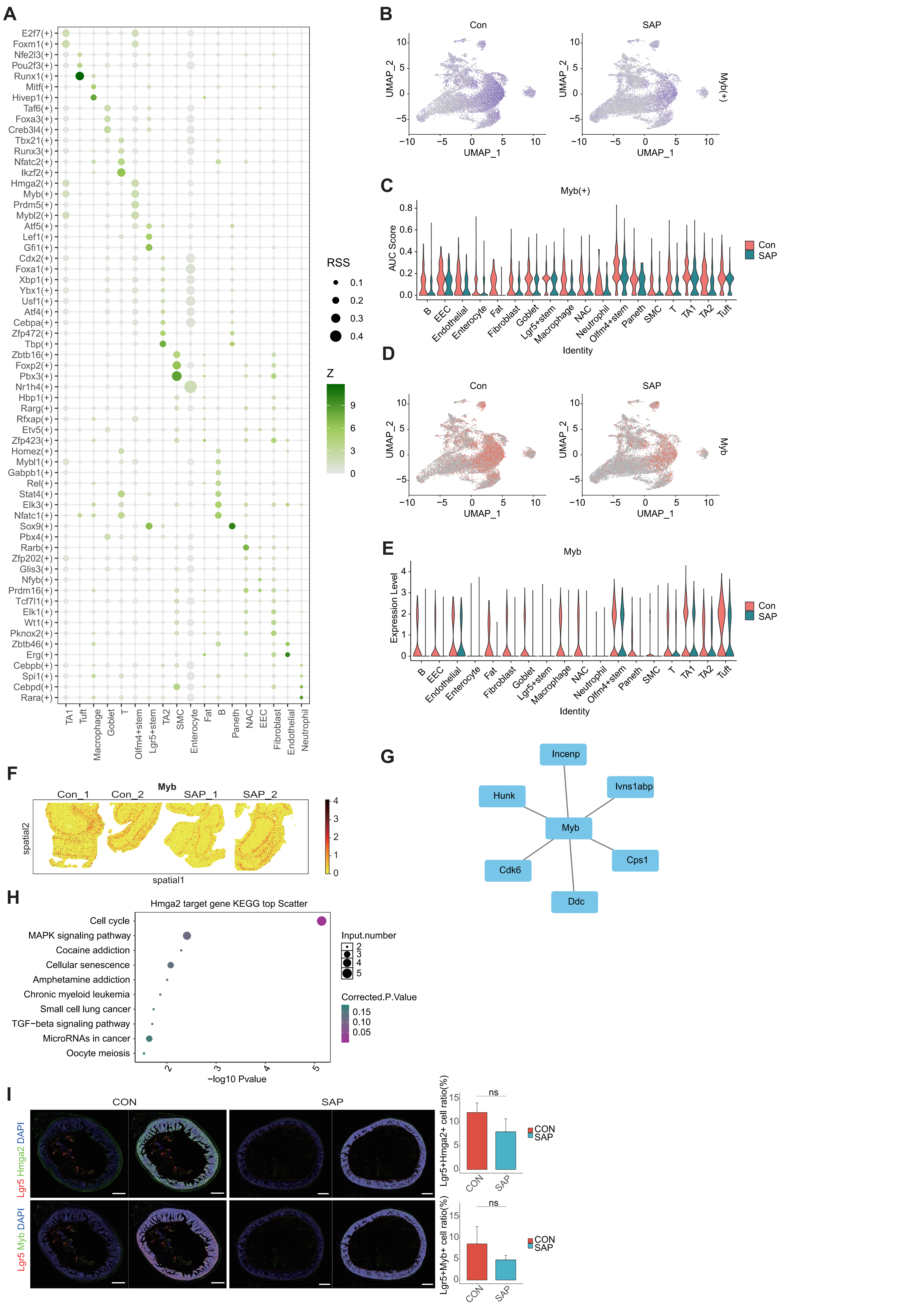

Supplement: Supplementary Figure 8 — Transcription factor regulon analysis of different cell types. (A) Dot plot showing specific transcription regulons in different cell types. (B) UMAP plot depicting the activation level of transcription regulon Myb(+) in different groups. (C) Violin plots depicting the activation level of transcription regulon Myb(+) of each cell type in different groups. (D) UMAP plot showing the expression levels of Myb in different groups. (E) Violin plot showing the expression levels of Myb in each cell type in different groups. (F) Spatial visualization of the expression levels of Myb in each sample. (G) Network of transcription regulon Myb(+). (H) Bubble plot showing the most enriched KEGG pathways of the target genes of transcription factor Hmga2 predicted by pySCENIC. (I) Representative immunofluorescence staining showing reduced expression of Hmga2 and Myb in Lgr5+ stem cells. Scale bars, 500 μm. Quantification was performed on 6 fields of view per group, derived from 3 independent animals per group. Data are presented as mean ± SEM. [file Image8.tif]
